# Investigating the collection and assessment of patient-reported outcome data amongst unplanned surgical hospital admissions: a feasibility study

**DOI:** 10.1186/s40814-015-0011-5

**Published:** 2015-05-09

**Authors:** John D Mason, Natalie S Blencowe, Angus GK McNair, Daniel J Stevens, Kerry N Avery, Anne M Pullyblank, Jane M Blazeby

**Affiliations:** 1 Centre for Surgical Research, School of Social and Community Medicine, University of Bristol, Canynge Hall, 39 Whatley Road, Bristol, BS8 2PS UK; 2 Nuffield Department of Surgical Sciences, University of Oxford, John Radcliffe Hospital, Oxford, Headington OX3 9DU UK; 3 Department of Surgery, North Bristol NHS Trust, Southmead Hospital, Southmead Way, Bristol, BS10 5NB UK; 4 Division of Surgery, Head and Neck, University Hospitals Bristol NHS Foundation Trust, Bristol Royal Infirmary, Marlborough Street, Bristol, BS2 8HW UK

**Keywords:** Emergency surgery, Feasibility, Methodology, Patient reported outcomes, Trial design

## Abstract

**Background:**

Randomised controlled trials (RCTs) in surgery can be challenging to conduct, and trials in the emergency surgical setting when patients have unplanned hospital admissions are particularly difficult. One area of challenge is capturing baseline patient-reported outcome (PRO) data. This study examined the feasibility and optimal methods for the collection of baseline and follow-up PRO data in the setting of unplanned surgical hospital admissions.

**Methods:**

Clinically stable adult patients with unplanned admissions through the day and night under the care of general surgeons at two acute NHS trusts were approached during working week days and asked to complete validated PRO measures (European Quality of Life-5 Dimension, Short Form-12, and Gastrointestinal Quality of Life Index) on admission and 6 weeks following discharge. Feasibility of PRO data collection was determined by the proportions of admitted patients eligible and recruited and by questionnaire-response rates at baseline and follow up. Reasons for non-recruitment and non-completion of questionnaires were sought and recorded.

**Results:**

There were 276 admissions, of whom 235 (85.1 %) were eligible. Reasons for ineligibility were the following: age under 18 years old (*n* = 5, 1.8 %), non-surgical presenting complaint (*n* = 6, 2.2 %) and clinical instability (*n* = 30, 10.9 %). One hundred and sixty-six patients (70.6 %) were recruited (98 female, 59.0 %); median age 53, range 19–100). Common reasons for non-recruitment included patients being discharged home before approached by researchers (*n* = 29, 12.3 %) or declining participation because they felt unwell (*n* = 15, 6.4 %). The most common reason for admission to the hospital was abdominal pain (*n* = 120, 72.3 % recruited patients), of whom 50 (30.1 %) required operative intervention. Baseline PRO data was obtained from 153 patients (93.3 %), and 74 (48.4 %) returned follow-up questionnaires.

**Conclusions:**

Collection of baseline PRO data amongst unplanned admissions in general surgery is feasible. Methods for optimising retention and follow up are needed.

## Background

Well designed and conducted randomised control trials (RCTs) in surgery are uncommon, and therefore surgical treatments are estimated to be half as likely to be based on evidence when compared with medical therapies [1]. This difference may be explained by the underlying challenges of designing and conducting RCTs in surgery, and surgeons often lack familiarity with these processes [2]. Specific challenges relate to the complexity of surgical interventions and how these are delivered and monitored in trials, as well as difficulties with recruitment and randomisation [1–4]. Furthermore, surgical trials often focus on outcomes relevant to surgeons (e.g. technical endpoints such as morbidity), and there is a lack of RCTs in surgery with high-quality patient-reported outcome (PRO) data [5, 6].

Emergency surgery represents a specific area of surgery where evidence from high quality RCTs is particularly lacking. In England, emergency general surgery represents approximately half of the general surgical workload and accounts for over 600,000 hospital admissions at a cost of 88 million pounds every year [7]. Despite this, the provision of emergency surgical care is considered suboptimal, with substantial variations in morbidity and mortality between centres [8]. Much research has therefore focused on improving care, but this is often of poor quality, comprising small retrospective case series within single centres. There are additional challenges to designing high-quality RCTs in emergency surgery as admission to the hospital is unplanned, and patients are acutely unwell making acquisition of informed consent for research and baseline data collection difficult. Furthermore, evidence of patients’ experiences of emergency surgical care is particularly lacking [7, 8]. PROs can be assessed using questionnaires known as patient-reported outcome measures (PROMs). Whilst these are designed to be completed by patients, this is difficult in emergency settings when many patients are admitted to the hospital unwell and in pain. Additionally, there may be insufficient time for patients to complete baseline questionnaires before urgent surgical intervention. The long term impact of emergency surgery on PROs is also needed to establish clinical effectiveness of treatments, but this can be difficult if patients have returned to normal activities and recovered from the acute illness before follow-up data is sought.

In some non-elective clinical settings including oncology [9] and paediatrics [10], methods for measurement of PROs have been established, but little is currently known about the feasibility of PRO data collection in emergency general surgery and optimal methods for achieving this. The aim of this study, therefore, was to examine methods for PRO data collection within a non-trauma emergency surgery setting.

## Methods

A prospective cohort feasibility study was performed within the surgical assessment units at two acute NHS trusts in the South West of England (one a university hospital and one a district general hospital) to determine the feasibility of the following: 1) recruitment and 2) collection of baseline and follow-up PRO data within a non-trauma emergency general surgery setting. The study received full ethical approval (REC reference number 13/SW/0028).

### Feasibility of recruitment

Patients over the age of 18 who were admitted with non-trauma emergency abdominal problems and managed by general surgeons were included. Excluded were patients without capacity to consent or those with immediate life-threatening conditions, defined as the following: 1) patients requiring immediate transfer to intensive care or theatre or 2) clinically unstable patients requiring ongoing resuscitation.

Eligible patients were approached after an initial clinical assessment in the surgical assessment unit and provided with a verbal outline of the study supported by a written information sheet. Written informed consent was gained after a consideration period of up to 1 h. Reasons for non-recruitment were recorded, and when patients declined recruitment, an explanation was noted if provided without direct questioning. The time taken to approach patients once admitted was recorded. Recruitment of eligible patients was undertaken from Monday to Friday and between 0800 and 1800 by two medically qualified research fellows (JM and DS) who did not form part of the emergency team or contribute to the clinical management of admitted patients. Attempts were made to recruit those patients admitted outside of these hours by approaching them when a member of the research team was next available.

To explore factors influencing feasibility, the following clinical data were collected from all patients: day and time of admission, reason for admission, final diagnosis and nature of treatment received. Additional socio-demographic data were recorded only from recruited patients (in accordance with research ethics guidelines) including age, sex, educational background, marital status and employment status. Admission and clinical data were tabulated separately for eligible and recruited patients and descriptive statistics used to compare groups. Feasibility of recruitment was determined by establishing the proportions of potentially eligible, approached and recruited patients.

### Feasibility of PRO data collection

Recruited patients were provided with paper copies of three PROMs representing the most commonly used measures in unscheduled gastrointestinal surgery [11]. The European Quality of Life Questionnaire (Euroqol) consists of the following two parts: 1) the European Quality of Life-5 Dimension (EQ-5D), a generic measure of health status that comprises five domains (mobility, self-care, usual activities. pain and discomfort and anxiety and depression) and 2) a global health visual analogue scale (EQ-VAS), which takes 5 min to complete [12]. The Short Form-12 (SF12) measures composite scores of physical and mental health and takes approximately 5–10 min to complete [13]. The Gastrointestinal Quality of Life Index (GIQLI) is a system specific PROM containing 35 questions relating to the gastrointestinal system and the impact of symptoms and treatment on individuals’ physical, emotional and social status. It takes approximately 5–10 min to complete [14].

Questionnaires were completed by the patients themselves without assistance from the investigators, except where they lacked the physical capacity to read or write. If required, questions were read aloud and responses recorded verbatim without interpretation by the researcher. Professional translation services were used when patients could not understand English and when available. When patients felt unable to complete all the questionnaires, a reduced number was offered in an attempt to capture at least some PRO data. If baseline PRO data was collected, follow-up questionnaires were posted to participants 6 weeks following discharge from the hospital. Non-responders received a single telephone reminder 2 weeks after the questionnaire was sent.

Feasibility of PRO data collection was determined by baseline and follow-up response rates. Reasons for providing patients with less than the full three PROMs were documented. Responses were defined as “complete” when patients returned all provided questionnaires, “incomplete” if fewer questionnaires were returned then provided and absent where no data was provided.

## Results

### Feasibility of recruitment

**Fig. 1:**
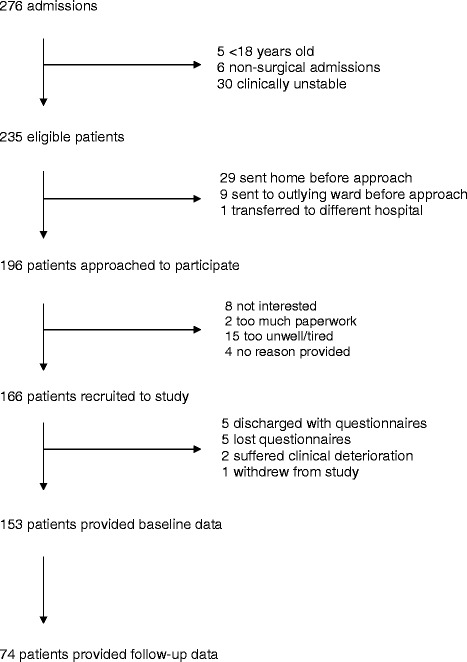
Flow diagram of patients through the study

A total of 276 patients were admitted to the surgical assessment units during the 7-week study period, of which 235 (85.1 %) were eligible. Of those ineligible, five (1.8 %) were under the age of 18, six (2.2 %) had a non-surgical reason for admission to the hospital and 30 (10.9 %) were clinically unstable. Some 166 (70.6 %) provided informed consent and were recruited (Fig. 1). The most common reasons for non-recruitment were patients discharged home before being approached by the researcher (*n* = 29, 12.3%), or declining study participation because they felt too unwell (*n* = 15, 6.4 %). Of those discharged before being approached, 14 (6.0 %) were at the weekend. The median time to approach patients once they had undergone an initial clinical assessment was 695 min (inter quartile range = 364–915 min).

Clinical and socio-demographic data are presented in Tables 1 and 2, respectively. Characteristics of eligible and recruited patients were similar. The majority of recruited patients were female (98, 59.0 %), and the median age was 53 (range 19–100). Abdominal pain was the most common reason for referral (120, 72.3 % recruited patients). Of the recruited patients receiving operative treatment (50, 30.1 %), appendicectomy was the most commonly performed procedure (14, 8.4 %). Patients were most commonly admitted between 1700 and 0800 h (107, 64.5 % recruited patients) and on weekdays (142, 85.5 % recruited patients).

**Table 1:** Clinical information of eligible and recruited patients

	Eligible patients *n* = 235 (%)	Patients recruited *n* = 166 (%)
Day of admission		
Monday–Friday	200 (85.1)	142 (85.5)
Saturday–Sunday	35 (14.9)	24 (14.5)
Time of admission		
08:00–17:00	85 (36.2)	59 (35.5)
17:01–22:00	88 (37.4)	61 (36.7)
22:01–07:59	59 (25.1)	46 (27.7)
Unknown	3 (1.3)	0 (0)
Presenting complaint (%)		
Abdominal pain		
Upper	34 (14.5)	26 (15.7)
Lower	51 (21.7)	38 (22.9)
Unspecified	83 (35.3)	56 (33.7)
Painful lump/hernia/abscess	19 (8.1)	15 (9.0)
Rectal bleeding	17 (7.2)	14 (8.4)
Abdominal distention	16 (6.8)	9 (5.4)
Abdominal mass	3 (1.3)	2 (1.2)
Jaundice	3 (1.3)	2 (1.2)
Other	9 (3.8)	4 (2.4)
Final diagnosis (%)		
Non-specific abdominal pain	52 (22.1)	30 (18.1)
Appendicitis	19 (8.1)	16 (9.6)
Upper gastrointestinal		
Pancreatitis	20 (8.5)	12 (7.2)
Billary	23 (9.8)	20 (12.0)
Other	12 (5.1)	9 (5.4)
Colorectal		
Diverticular disease	17 (7.2)	14 (8.4)
Other	32 (13.6)	23 (13.9)
Abdominal wall hernia	12 (5.1)	12 (7.2)
Other (e.g. urological and gynaecological)	48 (20.4)	30 (18.1)
Treatment Interventional	60 (25.5)	50 (30.1)
Appendicectomy	18 (7.7)	14 (8.4)
Hernia repair	9 (3.8)	9 (5.4)
Cholecystectomy	7 (3.0)	7 (4.2)
Colectomy	4 (1.7)	2 (1.2)
Laparoscopic drainage/drain placement	4 (1.7)	3 (1.8)
Diagnostic laparotomy/laparoscopy	3 (1.3)	2 (1.2)
Incision & drainage of abscess	3 (1.3)	2 (1.2)
ERCP	9 (3.8)	8 (4.8)
Radiology drainage	3 (1.3)	3 (1.8)
Non-interventional	175 (74.5)	116 (69.9)

*ERCP* endoscopic retrograde cholangiopancreatography

**Table 2:** Socio-demographic details of recruited patients

	Patients recruited *n* = 166 (%)
Female	98 (59.0)
Median age (range)	53 (19–100)
Marital status*	
Married	72 (46.5)
Single	31 (20.0)
Widowed–widower	25 (16.1)
Divorced	16 (10.3)
Co-habiting	11 (7.1)
Educational background*	
None	35 (22.6)
GCSEs	60 (38.7)
A-level	20 (12.9)
University degree	22 (14.2)
Vocational qualification	18 (11.6)
Employment status*	
Full-time	61 (39.4)
Retired	58 (37.4)
Part-time	16 (10.3)
Unemployed–sickness	9 (5.8)
Housewife/husband	4 (2.6)
Unemployed–seeking work	3 (1.9)
Other	4 (2.6)

*Socio-demographic information obtained from 156 patients. Of the ten missing patients; four were discharged before information could be obtained, two were transferred to a different department before information could be obtained, three did not provide information for clinical reasons and the reason was unknown for one patient

### Feasibility of PRO data collection

One hundred and sixty-four (98.8 %) patients were provided with at least one PROM at baseline. All three questionnaires were provided to 140 (84.3 %) patients, with two and one questionnaires provided to two (1.2 %) and 22 (13.3 %) patients, respectively (Table 3). Reasons for not providing patients with all three PROMs included an inability to complete the questionnaire even with physical help (*n* = 12), non-English-speaking and translator unavailable (*n* = 2), time constraints (*n* = 2) and pain (*n* = 8). Two patients were not provided with any questionnaires after recruitment due to clinical deterioration and rapid transfer to theatre.

**Table 3:** Number of PROMS provided and returned at baseline and follow up

	Baseline *n* = 166 (%)	Follow-up *n* = 153 (%)
Number of PROMs provided		
0	2 (1.2)*	0 (0)
1	22 (13.3)	0 (0)
2	2 (1.2)	0 (0)
3	140 (84.3)	153 (100.0)
Number receiving each PROM		
EQ5D	159 (95.8)	153 (100.0)
SF12	142 (85.5)	153 (100.0)
GIQLI	146 (88.0)	153 (100.0)
Number of patients completing all questionnaires provided		
Complete	149 (89.8)	74 (48.4)
Incomplete	4 (2.4)	0
Absent	13 (7.8)^**†**^	79 (51.6)
Number completing each PROM		
EQ5D	149 (89.8)	74 (48.4)
SF12	130 (78.3)	74 (48.4)
GIQLI	133 (80.1)	74 (48.4)

*one patient suffered deterioration in clinical condition, and one patient went to theatre

^**†**^five patients lost forms, five patients were discharged with forms, and one stated that he/she wanted to withdraw from the study

The overall baseline response rate was 92.1 % (*n* = 153, Table 3), with the majority of responses “complete” (149, 89.8 %), with a few “incomplete” (4, 2.4 %). Some 13 (7.8 %) patients did not return baseline questionnaires. Reasons for missing baseline data included lost questionnaires (*n* = 5), patients being discharged before questionnaire collection (*n* = 5) and withdrawal from the study (*n* = 1). Follow-up questionnaires were sent to the 153 patients with baseline data, and all were provided with the three PROMs (Table 3). Despite phone call reminder, the follow-up response rate was 48.4% (*n* = 74). All returned questionnaires were “complete”.

Socio-demographic details of individuals only providing baseline PRO data were similar to those who also completed follow-up PRO questionnaires (Table 4).

**Table 4:** Socio-demographic details of patients that provided baseline PRO data only compared with those who also providedfollow-up PRO data

	Patients providing baseline PRO data only *n* = 79 (%)	Patients providing baseline and follow-up data *n* = 74 (%)
Female	47 (59.5)	43 (58.1)
Median age (range)	55 (19–100)	61 (19–97)
Marital status		
Married	38 (48.1)	33 (44.6)
Single	17 (21.5)	15 (20.3)
Widowed-widower	11 (13.9)	13 (17.6)
Divorced	7 (8.9)	8 (10.8)
Co-habiting	6 (7.6)	5 (6.8)
Educational background		
None	20 (25.3)	13 (17.6)
GCSEs	29 (36.7)	32 (43.2)
A-level	9 (11.4)	11 (14.9)
University degree	10 (12.7)	12 (16.2)
Vocational qualification	11 (13.9)	6 (8.1)
Employment status		
Full-time	38 (48.1)	28 (37.8)
Retired	23 (29.1)	27 (36.5)
Part-time	6 (7.6)	10 (13.5)
Unemployed–sickness	6 (7.6)	4 (5.4)
Housewife/husband	3 (3.8)	1 (1.4)
Unemployed–seeking work	1 (1.3)	2 (2.7)
Other	2 (2.5)	2 (2.7)

## Discussion

This study assessed methods for patient recruitment and PRO data collection at baseline and follow up in an emergency general surgical setting. Over 70 % of eligible patients were successfully recruited and characteristics between those eligible and recruited were similar, suggesting that recruitment bias was low. Many patients were admitted overnight or on weekends, but high recruitment was achieved despite researchers working only weekdays, and the median time from admission to approach by the researcher was less than 12 h. Baseline PRO data collection was similarly strong, with over 90 % of patients returning at least one questionnaire. Follow-up response rates were lower (48.4 %), which may reflect some of the challenges of conducting research within an emergency context. Despite this, the study has demonstrated that weekday working can ensure high quality PRO data collection in an emergency surgical setting and is feasible. As such, it is recommended that future trials and prospective studies in this setting measure PROs in addition to clinical endpoints, but further work is needed to improve follow-up response rates.

This study is novel, and it has not been possible to identify other research exploring methods and feasibility of PRO data collection in unplanned general surgical admissions. A literature review found that existing RCTs investigating PROs in non-trauma emergency surgery were often at a high or unclear risk of bias [11]. There was often incomplete or missing data relating to overall rates of recruitment, and where this information was provided, successful recruitment ranged from 17.7–83.0 % [15–21]. Furthermore, there were weaknesses and inconsistencies in PRO reporting. Amongst the six identified RCTs, only one described baseline response rates (84 %) [22]. Similar studies have been conducted in other areas of emergency medicine. For example, a cross-sectional study of emergency medical admissions assessed health utility using the EQ-5D questionnaire and achieved a response rate of 47.7 % (2488/5760) using a postal questionnaire [23]. Another postal study used the SF-36 to assess quality of life following traumatic vascular injury with a response rate of 21.0 % (214/1018) [24]. These data are comparable to those found in the current study. No studies were identified which specifically addressed the key difficulty of baseline data collection and this may warrant consideration in order to inform future studies in this area.

The results of this work will assist in the design and development of future trials in emergency surgery. It shows that data can be collected effectively, with adequate response rates, when staff is available only on weekdays. It may not, therefore, be necessary to employ overnight or evening staff which could reduce research costs. There are, however, several limitations to this study. Data was collected by two medically qualified researchers, which may be prohibitive in future RCTs due to high financial costs. In elective surgery, baseline and follow-up data collections rates of 75.1 % and 59.6 %, respectively, have been achieved using nursing and administrative staff [25]. Whether this would translate into similar success in emergency surgery is unknown and warrants further investigation. Although efforts were made to expose recruitment bias by comparing characteristics of eligible and recruited patients, ethical considerations limited data collection for non-consenting patients. Although appropriate, this weakens the conclusion that recruitment was unbiased because key unmeasured characteristics could not be compared. Furthermore, clinically unstable patients were specifically excluded from this study. Targeting this group of patients will always be complex, and experiences from previous trials in acute medicine and trauma highlight the challenges of baseline data collection and the acquisition of informed consent prior to this [26, 27]. In addition, several patients were not provided with the full complement of questionnaires at baseline. This was planned so that recruitment and data collection was maximised when patients may otherwise have declined due to high questionnaire burden.

The response rate of 48.4 % may reflect the fact that postal questionnaires tend to have lower response rates than other data collection methods [28]. Telephone and face-to-face interviews may be used to collect data, however, this can be impractical and a very expensive method to use in large trials. There is a need to explore this further and establish if other methods (e.g. the use of incentives, more phone calls, interviews or appointments) can improve follow-up response rates. Another factor influencing the response rate for follow-up data may be the lack of assistance in completing the postal questionnaires. During collection of baseline data in the hospital, researchers provided patients with help (for example, reading questions aloud, marking answers and providing translation services). It is unknown whether such assistance was accessible to patients following discharge from the hospital.

Finally, the proportion of patients that underwent surgery in this study appears low. However, it is not uncommon for patients admitted under the care of surgeons to receive non-surgical treatment for conditions such as pancreatitis or diverticulitis. Such patients are often similarly unwell as those receiving surgery and this should not, therefore, affect the generalizability of this study. Furthermore, rates of surgery between the two study centres were similar.

A number of areas of future research need to be conducted before PRO evaluation within emergency surgery can be optimised. The EQ5D, SF12 and GIQLI were chosen because they are the most commonly used validated PROMs in studies investigating outcomes in non-trauma emergency surgery [11]. However, these have not been validated in an emergency surgery population, and may therefore be inappropriate or irrelevant when considering specific issues in this context. Disease-specific measures may need to be developed in order to fully evaluate emergency surgical interventions. Future research also needs to address the issue of heterogeneity in outcome measurement amongst emergency surgical studies. One solution would be to develop a core outcome set for emergency surgical studies. Core outcome sets represent the minimum outcomes to be measured in trials in specific contexts or disease areas, to reduce outcome reporting bias and facilitate meta-analysis [29]. They are usually developed by obtaining consensus between all stakeholders and crucially include patients’ views. Methods to define core outcome sets are now established [29, 30], and many are being developed in a range of clinical contexts [31–33]. Furthermore, research funding bodies are beginning to mandate their use when available. For robust evaluation of emergency surgery to continue, it is imperative that a core outcome set is developed.

## Conclusions

Patient recruitment and collection of baseline PRO data within the challenging environment of emergency non-trauma general surgery is achievable. Further work is required to optimise the collection of follow-up PRO data in this patient cohort. Once future research defines which PROs should be measured, robust clinical trials can be designed to evaluate emergency surgery with the same robust methodology that is applied in other specialties.
